# Supplementary material for: Evaluation of Functional Quality of Maize with Different Grain Colors and Differences in Enzymatic Properties of Anthocyanin Metabolism
Source: Foods. 2025 Feb 7;14(4):544. doi: 10.3390/foods14040544 (PMC11854767; doi:10.3390/foods14040544)
Supplement: Supplementary file 1 [file foods-14-00544-s001.zip › foods-3411341-supplementary.pdf]

Supplementary Table S1 Contents of soil basic nutrients

Soil layer (cm)	Availabile N (mg/kg)	Availabile P (mg/kg)	Availabile K (mg/kg)	Organic matter (g/kg)
0-5	96.88	66.35	293.87	14.84
5-10	42.16	22.65	195.74	16.40
10-20	38.15	12.27	138.37	14.35

Supplementary Table S2 Time of artificial pollination and sample collection

Pollination time	Pollination variety	Sample collection time			
		14 DAP	18 DAP	22 DAP	26 DAP
July 26th	Jindannuo41, Jinong7, Jinnuo10	August 9th	August 13th	August 17th	August 21st
August 3rd	Jinnuo18, Wannuo2000, Jinnuo20	August 17th	August 21st	August 25th	August 29th

Supplementary Table S3 Eigenvalues of correlation matrix and eigenvectors of corresponding matrices for different varieties of waxy maize at four stages

Primary Component		Principal component number		
		1	2	3
Eigenvalue		3.459	1.697	1.347
Percentage of Variance (%)		38.431	18.860	14.969
Cumulative (%)		38.431	57.291	72.260
Load factors	Carotenoid	-0.205	0.166	0.662
	Soluble sugar	0.418	-0.030	0.175
	Vitamin C	0.424	-0.057	-0.152
	Anthocyanin	0.245	0.316	-0.538
	Fe	0.347	-0.395	0.292
	Mn	0.211	0.537	0.276
	Zn	0.317	0.447	0.197
	Cu	0.481	-0.048	0.015
	Ca	0.216	-0.472	0.134

Supplementary Table S4 Principal component score for different varieties of waxy maize at four stages

	PC1 (38.4%)	PC2 (18.9%)	PC3 (15.0%)	PC	Ranking
J18-S1	0.25	-1.2	0.22	-0.1	14
W2000-S1	2.68	-1.52	0.32	0.79	5
J41-S1	2.8	-0.59	1.27	1.16	3
J7-S1	1	-1.83	0.67	0.14	10
J10-S1	3.61	0.76	0.07	1.54	1
J20-S1	2.75	0.65	-0.46	1.11	4
J18-S2	-2.74	-0.64	-0.06	-1.18	24
W2000-S2	-0.91	0.72	-0.64	-0.31	16
J41-S2	-1.38	1.89	1.93	0.12	11

J7-S2	-2.23	0	0.22	-0.83	21
J10-S2	-0.19	1.79	-0.32	0.22	7
J20-S2	2.7	1.88	0.47	1.46	2
J18-S3	-0.98	-3.36	-0.36	-1.06	23
W2000-S3	0.51	-1.1	0.54	0.07	12
J41-S3	-0.74	1.03	2.13	0.23	6
J7-S3	-1.75	-0.45	1.2	-0.58	18
J10-S3	0.77	0.39	-1.14	0.2	9
J20-S3	1.15	-1.23	-1.67	-0.04	13
J18-S4	-1.91	0.03	-0.78	-0.84	22
W2000-S4	-1.99	0.53	-1.03	-0.82	20
J41-S4	-1.44	0.92	1.21	-0.2	15
J7-S4	-1.64	-0.77	0.48	-0.7	19
J10-S4	0.59	1.03	-1.4	0.21	8
J20-S4	-0.91	1.09	-2.87	-0.57	17
**Stage**					
S1	2.18	-0.62	0.35	0.77	1
S2	-0.79	0.94	0.26	-0.09	2
S3	-0.17	-0.79	0.12	-0.2	3
S4	-1.22	0.47	-0.73	-0.49	4
**Variety**					
J18	-1.35	-1.29	-0.24	-0.8	6
W2000	0.07	-0.35	-0.2	-0.07	4
J41	-0.19	0.81	1.63	0.33	3
J7	-1.16	-0.76	0.64	-0.49	5
J10	1.19	0.99	-0.7	0.54	1
J20	1.42	0.6	-1.13	0.49	2
